# Nested Houses: Domestication dynamics of human–wasp relations in contemporary rural Japan

**DOI:** 10.1186/s13002-017-0138-y

**Published:** 2017-02-08

**Authors:** Charlotte L. R. Payne, Joshua D. Evans

**Affiliations:** 1 Conservation Science Group, Department of Zoology, David Attenborough Building, New Museums Site, University of Cambridge, Cambridge, CB2 3QY UK; 2 Department of History and Philosophy of Science, Free School Lane, University of Cambridge, Cambridge, CB2 3RH UK

**Keywords:** Domestication, Ecology, Edible insects, Japan, Traditional food, Vespula, Wasp

## Abstract

**Background:**

Domestication is an important and contested concept. Insects are used as food worldwide, and while some have been described as domesticated and even ‘semi-domesticated’, the assumptions and implications of this designation are not clear. The purpose of this paper is to explore these aspects of insect domestication, and broader debates in domestication studies, through the case of edible wasps in central rural Japan.

**Methods:**

Both authors conducted ethnographic fieldwork with communities in central rural Japan. Fieldwork comprised participant observation, semi-structured interviews, quantitative surveys and a review of resources including the personal and public records of wasp collectors.

**Results:**

The practice of keeping wasps in hive boxes has historical roots and has changed significantly within living memory. Current attempts to further develop the practice involve collectors’ great efforts to keep new queens during their hibernation. Collectors have also tried, still without success, to keep wasps living within a human-made enclosure for their entire life cycle. These and other practices are costly in both time and money for collectors, who emphasise enjoyment as their primary motivation. At the same time, they also engage in practices such as pesticide use that they recognise as damaging to wasp ecology.

**Conclusions:**

These practices can be understood to some extent in domesticatory terms, and in terms of care. We develop a framework for understanding domesticatory practices of insect care, discuss how this case contributes to ongoing debates within domestication studies, and recommend further research to be pursued.

## Background

We (Charlotte Payne, hereafter CP and Joshua Evans, hereafter JE) have seen and joined in with many wasp-related activities—chasing, collecting, harvesting, eating—in rural Japan. Together we have discussed wasp-keeping, overwintering, and their ecological implications, at length. The catalyst for these discussions is best traced back to a wintry morning in 2013, when CP was given the opportunity to see the practice of 越冬させる—‘ettō saseru’, or overwintering—first-hand. We thus begin our paper with CP’s first-person account of that day.

***23.12.2013, Asuke, Japan.***

*Hori-san and I climbed a steep driveway and arrived at a beautiful wooden house. Goto-san emerged. He lived alone, Hori-san explained, ever since his wife’s death a few years before. After a mumbled greeting, Goto-san ambled down a narrow path that curved around the back of his house, leading to a small shed with a wide front window. To the trained eye, this shed was instantly recognisable as a hebo (wasp) house. Goto-san opened the shed door. The warmth inside, maintained by a small fan heater in one corner, was a welcome respite from the cold morning air.*

*“The warmth keeps them active. See—these two are still inhabited.”*

*Of the eight hive boxes that lined the wall of the shed, six were no longer in use, with their entrances blocked; but at the openings of the other two, the occasional wasp came and went.*

*Goto-san took out a rectangular plastic container and placed it on a narrow wooden work bench. Then he pulled out a small stepladder and ascended until his head rose into the eaves of the small house. There, dozens of thin tubes lay across the beams of the roof. Some were bamboo; others plastic pipe. He began to take the tubes out one by one and peer into them. The first few he took he replaced, but the next he held and began to descend the ladder. He brought it over—inside, the tiny silhouette of a wasp was clearly visible, curled against the tube’s curve.*

*He took the tube, placed one end into the container, and tapped the other end until the hibernating wasp fell out onto the plastic. Then, he went back up the ladder for more.*

*Eventually, the container was filled with tiny curled-up wasps. Some had begun to sleepily unravel, but most lay dormant. The next job, he explained, was to sort the wasps. Some, he pointed out, were smaller males—destined to die in a few days. Others, however, were larger females, each a potential new queen who might found a colony the following year.*

*He took out another box, homemade of wood, and placed it on the worktop. It contained dry leaves, and a small saucer with a single leaf of Chinese cabbage. Dry leaves mimic the forest floor where the queens would usually hibernate, and the cabbage is for moisture, he explained. The box had a lid with tiny window and a tube for air. This would be their home until the following spring.*

*Using a pair of tweezers, he gently took each female wasp and placed it into the box, counting as he did so. When he had transferred one hundred wasps, he closed the box and labelled it—with today’s date and the number of wasps inside.*

*“He does this every morning,” Hori-san said to me with clear admiration. “Show her where you keep them,” he suggested to Goto-san, who obligingly walked back up the path to his garage, carrying the box. Inside, he pulled a tarpaulin from a large mound. Beneath the tarpaulin were a couple of heavy blankets. “The blankets keep them warm,” Hori-san explained. And beneath the blankets were the boxes: at least twenty of them piled carefully and systematically, each neatly labelled, and each containing one hundred potential wasp queens.*

CP’s morning in Goto-san’s shed and other subsequent experiences became central to our discussions of wasp care. We agreed that this practice of overwintering seemed to have a different significance from the other wasp-related activities. It represented a deeper kind of attention, a closer relationship with the rhythms of the wasps’ life cycle. It opened up a space in which both humans and wasps might affect each other and their surrounding ecology in more profound ways. Had we encountered a kind of selection, even a domestication, unfolding at the human timescale? What exactly would it entail to ‘domesticate’ a wasp, and how long might it take? Was this even a goal of the practices we observed and participated in? After further fieldwork, reflection and discussion, we decided these questions were worth deeper consideration.

### Domestication

#### What is it?

‘Domestication’ is notoriously difficult to define [1–4]. Scholars who work on domestication agree that it involves both biological and socio-cultural factors that are inextricably intertwined, yet most favour one ‘side’ of the relationship [5, 6]. Thus, studies of domestication may challenge and/or reinforce the dichotomy of biological nature and human culture [7–9].

We define domestication as ‘a process of hereditary reorganisation of organisms into new forms, according to [certain organisms’] interests,’, modified from Anderson [6].

#### How do we know it?

The processes and concept of domestication are studied across a wide range of disciplines. Yet since most such studies associate domestication’s processes primarily with agricultural revolutions, they engage with data primarily from the past. Here, we offer an ethnographic approach using data from recent decades. We argue that domestication processes can be investigated as they unfold at an observable time-scale, and that this approach can also help to bridge the divide between natural and social sciences in domestication studies [5].

#### What are the key debates?

Many debates in domestication studies revolve around the following issues:

- whether domestication emerged because of surplus or scarcity, or both [10–13]
- whether agencies in domestication processes are symmetrical or asymmetrical [14, 15];
- whether domestication requires deliberation/intentionality [16];
- whether domestication necessarily involves humans [17–19];
- whether domestication necessarily involves multiple species [20, 21];
- whether domestication entails progression or teleology [3, 22–24];
- whether domestication entails a set of universal conditions that identifies all domesticates regardless of context; and
- whether domestication presumes a converse state in which organisms are not already participating in mutual hereditary reorganisation [25, 26].

We return to these debates in the Discussion.

### Edible insects

#### The cultivation and domestication of edible insects

The silkworm (*Bombyx mori)* and the honeybee (*Apis mellifera)* have been identified as the only edible domesticates in the insect kingdom whose domestications predate written history [27, 28]. Other insects including *Dactylopius* spp., *Ericerus pela*, *Kerria* sp. and *Llaveia axin*, however, were also domesticated for their use in the production of dye, shellac and wax [29, 30], and yet others were domesticated for medicinal purposes [31]. Similarly, enclosed farming systems have been developed for other insects in more recent years. These include insects used for scientific purposes, such as the fruit fly [32], but also insects used as human food and animal feed, such as crickets, which are now farmed worldwide [33, 34].

Mutually supportive insect–human relationships are also recognised extensively worldwide. Many of these involve humans’ manipulation of the environment to increase the insects’ yields and to ensure their long term use as food. Key examples are outlined in Van Itterbeeck and Van Huis’ review article on the subject [35], and include aquatic Hemiptera in Mexico, palm weevil larvae (*Rhynchophorus* spp.) in Papua New Guinea, and bardi grubs in Australia [36]. There are also recent examples of the ongoing development of farming systems for edible insects, such as the mopane caterpillar [37, 38] and several species of wild silkworm in Asia and Africa [39–42].

It is this latter group of insect–human relations—environmental manipulation to increase yields—that we consider most appropriate for understanding human–wasp relations in Japan. The human–wasp relationship has been described in the literature: Nonaka [43] reports that people place young nests into hive boxes and provide them rich food in order to create larger nests for eventual consumption. Yet there is no mention of any institutional program aimed at domesticating wasps, as is the case for crickets and mopane caterpillars. Human–wasp relations in Japan thus provide an intriguing case of rapid and bottom-up domesticatory practices in recent history.

#### Social wasps as food

**Fig. 1:**
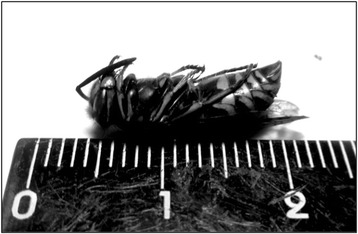
*Vespula* spp.

Scholars working across five continents—North and South America, Africa, Oceania and Asia—have documented long traditions of collecting social wasps’ nests in order to harvest larvae for food [44]. In Japan, the first known historical record of social wasps used as food lists four species, and dates from 1715; a further record from 1803 lists three species [45]. Figure 1 shows the most commonly collected species of edible wasp in contemporary Japan, the *kuro-suzume-bachi* (literally, the ‘black wasp’). This names refers to two species, *Vespula flaviceps* and *Vespula shidai* [46]*. V. shidai* are more prevalent in the north while *V. flaviceps* are more prevalent in the central region of the country [47].

In 1986, wasps were collected and consumed in 42 out of 47 Japanese prefectures [48]. An overall decline in insect consumption in Japan suggests that the prevalence of this practice may have decreased considerably in recent decades [49]. In some areas nests were collected from the *yama* (山), the forested mountain landscape, early in the season and transported to a location near the home, ensuring stewardship of the nest and its protection from natural predators [48]. It is unclear when this practice began, how widespread it was, and who the main ‘owners’ of nests were.

Overall, historical records suggest that while wasp consumption was historically practiced in many locations in Japan, nest transportation and stewardship was not a common practice.

#### The life cycle of a *Vespula flaviceps/shidai* colony

**Fig. 2:**
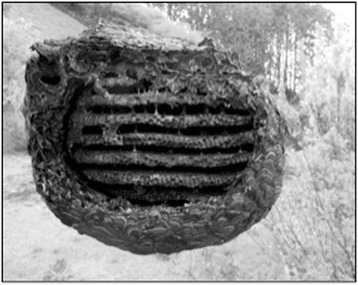
A *Vespula* spp. nest, with the surrounding material removed to show the layers. This nest had been reared in a hive box over the summer and left in the hope of ensuring there would be enough new queens who hibernated and successfully made nests the following year

The lifespan of a *V. flaviceps/shidai* colony in a temperate climate is annual. Only the gynes (the new queens; 新女王蜂、*shin-jo-ō-bachi*, in Japanese) survive the winter months. They emerge from their solitary hibernation during early May and seek a site at which to build their nest underground. They then lay eggs, which when fertilised hatch into diploid females (workers or new queens) and when unfertilised develop into haploid males. Workers help build the nest, and collect food to nurture new larvae throughout summer (June-October). The nest is largest during November. After this point, the temperature drops, and the queen lays eggs which will hatch into reproductive males and new queens. New queens mate with several males before hibernating, and store the sperm of each of these males inside their bodies [50]. When they lay eggs the following year, these eggs represent several patrilines, ensuring genetic diversity within the colony. Figure 2 shows a nest after the new queens have left to hibernate, with the outer layer removed to reveal the internal structure.

#### Research questions

Our research questions look at the (1) practices and attitudes, (2) historical developments, (3) investments, and (4) motivations in wasp–human relations in contemporary Japan:

1. *What are the prevalent practices and attitudes associated with wasp collection and consumption?*
2. *What have been the key developments in wasp–human relations?*
3. *How much time do people in contemporary Japan devote to finding, collecting and keeping wasps, and how many wasp nests do they keep per person?*
4. *What reason do people give for starting to keep wasps, and what do they do with their harvest?*

## Methods

The data described here were collected over an 18-month period from May 2013 to December 2014, encompassing two wasp seasons. CP undertook participatory fieldwork in Kushihara, Gifu prefecture, for the duration of this period; JE spent one week undertaking participatory fieldwork in Kushihara during November 2014. CP conducted informal and semi-structured interviews with residents throughout this period, documented as field notes, photographs and audio. JE conducted informal interviews, documented as field notes, photographs, and film.

In 2013 and 2014, CP participated alongside villagers in all stages of wasp keeping described in this paper. In 2014, JE participated in nest harvesting, nest processing, and wasp cooking. Both authors were, therefore, ‘full-bodied participants’ [51] in wasp–human relations.

In 2013, CP distributed a questionnaire to people who keep wasps (*n* = 73) at three events celebrating the wasp harvest, in Kushihara (Gifu prefecture), Higashi-shirakawa (Gifu prefecture) and Ina (Nagano prefecture). This questionnaire asked respondents about the time they spend collecting wasps, the number of nests they collect, and how they divide up the wasp harvest. The majority of respondents were male (*N* = 70) and the average age of respondents was 64.7 years. The number of respondents by age, gender and event is shown in Table 1. CP also spoke to people at these events about their experiences with wasps, and made follow-up visits to speak in more depth with key interlocutors, most notably the leaders of wasp interest groups in different areas.

**Table 1:** Age and gender of questionnaire respondents

Wasp festival	Survey respondents	Female	Male	Average age (SD)
Kushihara	10	1	9	62.3 (±7.3)
Ina	26	0	26	64.3 (±11.2)
Higashi-shirakawa	37	2	35	65.3 (±12.1)
Total	73	3	70	64.68 (±11.2)

In addition to published literature in Japanese [48], we used historical records, contributed by the Japan Vespula Society, to inform our study: (1) documents recording the membership and activities of community groups attending the 全黒地蜂サミット (*zenkoku-jibachi-samitto*, or ‘national wasp summit’; summarised in Table 2); (2) documents recording the results of nationwide wasp nest contests held in Kushihara in 2008, 2009, 2011, 2012 and 2013; and (3) the personal records of an individual wasp collector, spanning 10 years from 2002 to 2012.

**Table 2:** Number of groups participating in each meeting described by the documents of the Japan Vespula Society

		Number of participating groups					
Document No	Year of meeting	Yamanashi	Gifu	Aichi	Shizuoka	Nagano	Total
1	1997	1	5	2	1	3	12
2	1998	2	5	3	1	4	15
3	1999	3	5	3	2	5	18
4	2000	3	5	3	2	5	18
5	2001	4	6	4	2	6	22
6	2002	5	8	4	2	9	28
7	2004	5	8	6	2	9	30
8	2006	6	8	6	3	9	32
9	2010	3	6	6	1	6	22

## Results

### What are the prevalent practices and attitudes associated with wasp–human relations?

#### Notes on the field setting

**Fig. 3:**
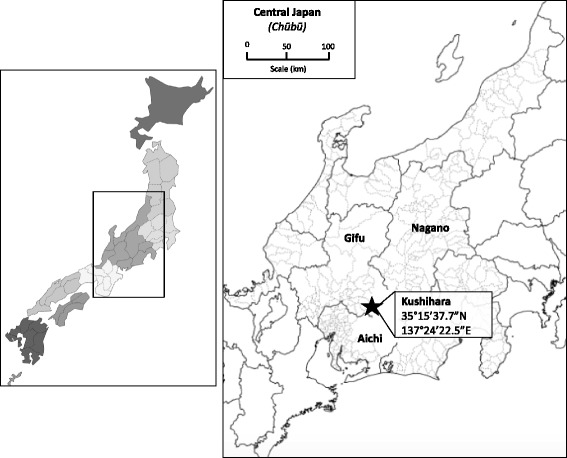
Map showing the location of Kushihara, in the Central (Chūbū) region of Japan

The majority of fieldwork was undertaken in Kushihara (串原), a village of 300 households in Gifu, or with communities in similarly-sized villages in Gifu, Aichi and Nagano (see Fig. 3 for a map of the region).

Kushihara lies at an average elevation of 434 metres above sea level in the Japanese alps. In summer (mid-June to mid-September), daytime temperatures reach 25–30 °C, dropping to 0–10 °C in winter (mid-December to late February). The landscape is hilly, with fields and terraced paddies interspersed with forested mountainsides. Forests are dominated by cedar and cypress, planted during the twentieth century for logging. Forestry has since declined, as prices have been undercut by imported wood, although the forests are now actively managed with a view to promoting biodiversity. We use the term ‘wild’ to distinguish between nests collected from the surrounding landscape and nests kept by carers, rather than to make any ontological claim about the landscape’s separation from human activity [52, 53].

The majority of interlocutors in this study were older than the 60-year retirement age. This generation has seen great social and economic change. Their childhood was dominated by wartime or postwar concerns of food rationing and inadequate harvests. Families had land, used for growing rice and vegetables, and all family members took part in agricultural labour. The work of planting and harvesting rice would often be shared by the community. These individuals were working adults during the bubble era of the late 1980s, and many are now affluent. They still own land and grow rice, but most rice fields are now planted and harvested communally, which is subsidised by the government [54]. Traditionally, in most households, the eldest male son inherits and lives in the family home with his wife and children, and is responsible for the upkeep of the house and land. Currently, young adults rarely follow this structure, however, and most village residents have children who live and work in cities.

#### Linguistic considerations

*Kuro-suzume-bachi* (クロスズメバチ) literally translates as ‘black sparrow wasp’: *kuro* is black, and *suzumebachi*, or ‘sparrow wasp’, is the name given to eusocial wasps. However, communities in which wasps are consumed as food often use a different, locally-specific, colloquial name. In Kushihara, this name is ヘボ (*hebo*). We cannot recall a single time that a Kushihara resident referred to these wasps by any other name unless in conversation with someone from outside the village. Other areas have similarly locally particular names, such as すがれ (*sugare*), and タカブ (*takabu*). The origins of these names are unclear. *Hebo*, for example, also means ‘clumsy’ in Japanese but people stressed that this was not the reason for the name. In a book [55], wasp collectors have been referred to collectively as はちたち (*hachi-tachi*), *hachi* meaning wasp and *tachi* denoting a familiar relation to a group—for example, the Japanese word for ‘friend’ and ‘friends’ is ともだち (*tomodachi*). However, we never heard this phrase used in speech.

The verb commonly used to describe the process of searching for the nests uses 追う (*ou*). The word implies chasing or following, but with an unusual pronunciation: ぼう (*bou*). We use the English verb ‘to chase’ here when referring to this process. The national television company NHK (*Nippon Hōsō Kyōkai*) has used ハンター (*hantā*, a phonetic adaptation of the English noun ‘hunter’) to describe those who ‘hunt’ for wasps. However, during fieldwork the Japanese word to ‘hunt’, 狩り (*kari*), was used only to describe searching for game animals such as wild boar. Colloquially, the verb やる (*yaru*)—the basic, informal form of ‘to do’—was also used to refer to those who collected and cared for wasps. For example, one could ask the question ‘ヘボをやるの?’ (*‘Hebo wo yaru no?’*) meaning literally ‘Do you/does (s)he do hebo?’.

The verb used to refer to a wasp landing on bait was 就く(*tsuku*), which can mean to ‘take’ or ‘get’ something (e.g. 職に就く, *shoku ni tsuku*, means to get a job). Calls of 就いた!就いたよ! (*‘Tsuita! Tsuita-yo!’*) communicated to other chasers that a wasp had ‘taken’ the bait. The white marker was a 目印 (*mejirushi*), a term also used to describe markers such as landmarks or a bookmark. The hive boxes were 巣箱 (*su-bako*), literally ‘nest box’. Small houses with multiple hive boxes inside them, often used for overwintering new queens, are usually referred to as ハウス (*hausu*, the phonetic Japanese version of the English ‘house’) or ヘボハウス (*hebo hausu*). Food given and meat used to bait wasps was 餌 (*esa*), which denotes feed for livestock or pets.

Placing the nests in hive boxes near to the home is referred to using the verb 植える (*ueru*), ‘to plant’. For example, the question いくつ植えたか (*ikutsu ueta ka?*) would be used to ask ‘how many [nests] did you plant?’ This may be because the practice originally began with people placing the nests in the soil near their homes.

#### Collecting, processing, and consuming wasps

A social wasp nest in a temperate climate is largest during late autumn. This is when wasp larvae were traditionally collected in central Japan, and the majority of people born and raised in each study site recall their fathers and grandfathers using this method to collect wasps. Some wasp collectors prefer to chase alone, but most chase in groups. In many of these groups, individual members are recognised for being skilled at executing different parts of the chase, and take on specific roles. Wasp chasing and collecting is regarded as ‘dangerous’ (危ない/*abunai*). This danger is considered to come from the act of navigating the *yama*. When asked, however, no interlocutor could recall a single example of a person who had been harmed while collecting wasps. Many had been stung, but without any break in activity, and stings to adult wasp chasers were not considered to require treatment.

The nest is located using a chasing process that incurs substantial time and energy costs: chasers leave meat or fish skewered on bamboo sticks in the forest to attract the carnivorous worker wasps. Several interlocutors reported that in the past, they would catch and skin frogs for this purpose. During our fieldwork, although some chasers used small, self-caught freshwater fish, raw squid purchased from the local supermarket was by far the most common bait (餌/*esa*), preferred for its pungent smell. When the chasers find a wasp consuming the bait, they offer it a small wasp-scale ball of meat attached to a thin cotton string with a white marker. Markers varied. Some chasers spent entire evenings carefully handcrafting markers using lightweight polypropylene or white plastic bags; others took cotton wool with them on the chase, and twisted it to make markers on the spot. If the wasp accepts this conveniently pre-cut morsel, it will carry it, with the marker, back to the nest. It is clearly visible and traceable as it flies through the forest. The task of offering the pre-cut meat to the wasps is often bestowed upon a certain person in the group, deemed most skilled. In the cases we observed or were told about in which couples chase wasps, the woman performed this role while the man followed the wasp through the forest.

The wasp chasers follow the wasp to locate the nest, usually requiring multiple attempts and constant communication between chasers. This is particularly important in areas of forest with steep slopes. For example, one person would follow the wasp to a certain point beyond which the wasp was no longer visible. Another chaser would go ahead to a location assumed to be in its flight path. The first person would call as the wasp passed, so that the second could spot it and continue to follow. A third person, if available, would do the same at an even further location. In some cases individuals could not see each other but communicated by shouting through the trees.

On one of her first wasp-chasing trips in Asuke, CP found herself, without being expressly told to do so, chasing the wasp in the foremost position of one such chain. On this occasion she successfully located a wasp nest. Consequently, when chasing wasps with the same group, she was encouraged by fellow chasers to continue at the head of the chain because of her ‘young’ eyes.

When the nest is found, chasers usually burn something to subdue the colony before digging out the nest. In our experience, this object would be a ping pong ball, or a はちとり (*hachi-tori*)—a purpose-made stick containing a zinc chloride smoke mixture known to sedate bees and wasps. Nests are dug in daytime, using a gardening trowel, and the person digging sometimes wears purpose-made protective clothing used by beekeepers. In the past, nests were dug after dark, without protective clothing, when wasps are less active.

The nest is then taken back to the human home and harvested. Larvae and pupae are individually removed from the comb cells with tweezers. Adults may also be collected. The wasps are then cooked for immediate consumption, or preserved using soy sauce and mirin (a cooking method known as *tsukudani*).

#### Relocation of wasps to human-made hive boxes

**Fig. 4:**
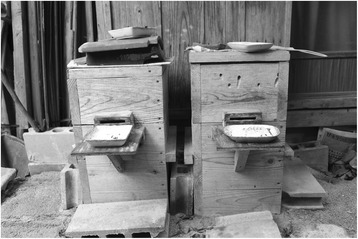
Two hive boxes. The roof is designed to ensure that the temperature inside the hives stays cool. A piece of meat is hanging by a wire in front of the entrances to the hives

Humans relocate wasp colonies in spring, when nests may be as small as a tennis ball. They make purpose-built cuboid nest boxes approx. 7.5-17.5 cm in each dimension. They use the same technique of baiting and following worker wasps to locate the nest. However, smoke is not used when the nest is dug up, as this could cause the queen to abandon the colony, threatening its survival. To keep as many mature wasps as possible inside the nest when it is dug up, the chaser will beat the ground above with a stick. This is said to alert the worker wasps to possible danger, causing them to retreat within the nest. The chaser then carefully digs out the nest and places it in the aforementioned small box, which is selected to best suit the size of the nest. Sometimes lengths of grass or folded paper are placed within the box to buffer it from any potential damage during transportation. Collectors attempt to collect any nearby adult wasps, which are also placed in the box. The wasp collectors then transport this box to the larger hive box, place food at the sealed entrances, and then remove the seals. This arrangement is shown in Fig. 4. If the nest falls apart when collected, it may be put back together using thin wire. Most wasp collectors feed their colony/-ies at least twice daily with a diet of raw flesh (often expensive meat such as raw chicken breast or liver, but less expensive cuts and river-caught small fish are also used) and rock sugar or sugar water, which helps the colony grow larger than it would otherwise. Collectors wait until late autumn to remove the fully-grown nest from the hive box and harvest the larvae within.

**Fig. 5:**
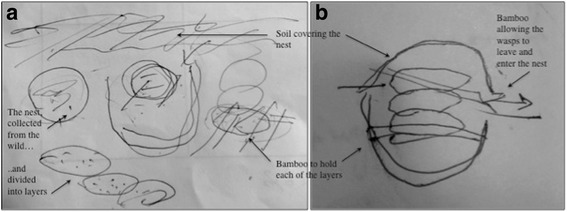
Two sketches, drawn independently by one man from Gifu prefecture (**a**) and one man from Aichi prefecture (**b**), showing how they remember keeping wasps as children. Annotated by the author, based on interview data

The historical practice of placing a nest in a hole in the ground is shown in Fig. 5. Two different people drew these sketches on independent occasions, and both recalled it mainly practised by young male children. Informants from other areas reported an absence of this activity among both adults and children. Success rates of wasp-keeping by this method were reportedly low: if a child attempted to raise three nests, for example, only one would survive to maturity. It is interesting to note that these boys are the same generation who were the main instigators of the most recent developments in wasp–human relations.

#### Wasp nest contest events

The first recorded wasp contest event took place in Shitara, Aichi prefecture, in 1990. The first nationwide wasp contest followed in Kushihara in 1993, where it has since been repeated on an annual basis. Similar events have also taken place in multiple locations in central Japan, and vary in their scale and atmosphere. During our fieldwork they were held on weekends from mid-October to mid-November. In 2013 there were 15 contests in central Japan over this period: three in Nagano, five in Gifu and seven in Aichi. CP attended two in Gifu (Kushihara, Higashi-shirakawa) and one in Nagano (Ina). In 2014 there were 13 contests; in 2015, 9. JE and CP attended one together in 2014 in Gifu prefecture (Kushihara). Each event shared the same basic structure: participants arrived in the morning with their hive boxes. These were taken to a location away from the main event area (e.g. inside a temporary greenhouse), and nests were removed from the boxes one by one and placed into labelled bags. These were then taken to the main event area for weighing and selling. Nests were sold at a price of up to 9000JPY (80USD) per kg. Visitors were able to purchase the nests themselves, and also a selection of foods with wasps made by local people. Examples included 五平餅 (*gohei-mochi*)*—* pounded sticky rice moulded into an oblong shape on a cedar stick, brushed with a sauce made by grinding together wasp larvae, soy sauce, miso, peanuts, sugar, and fresh ginger root, and grilled over wood embers—and 蜂の子ご飯 (*hachi-no-ko-gohan*), rice steamed with wasp larvae.

#### Communal processing with friends and family

**Fig. 6:**
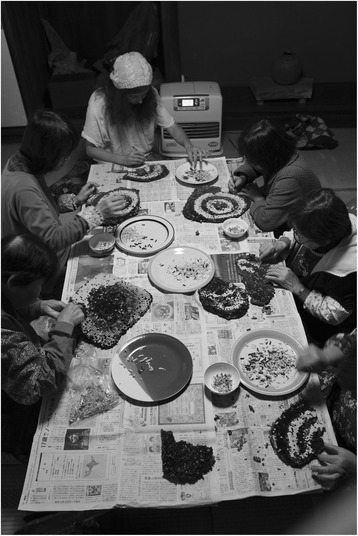
Removing the larvae and pupae one by one with tweezers

**Fig. 7:**
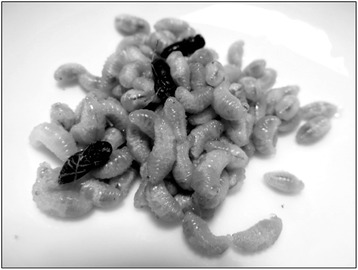
*Hachi-no-ko*, freshly-harvested wasp larvae

Processing the nests must take place fairly shortly after they have been removed from either a hive box or the forest, as each layer contains larvae and pupae that would die if not tended to. The layers of the nest are separated and the larvae and pupae are removed using tweezers, as shown in Fig. 6. Fresh larvae are shown in Fig. 7. The larvae, pupae, and adults are then cooked for immediate consumption or preservation, or frozen for future cooking. Methods vary slightly by region and household preferences, but the predominant preparation is 佃煮 (*tsukudani*): larvae and pupae are simmered with soy sauce and mirin, and sugar and/or sliced or grated ginger root to taste. *Tsukudani* wasps can be eaten immediately, often with rice, or preserved for weeks or months. Adult wasps are sometimes fried in oil and eaten immediately.

#### Environmental conservation

Informants who recalled harvesting wasps as children mentioned that wasps have declined and/or wasp distribution has changed within living memory. In the past, more wasp nests were found within the 里山 (*satoyama*) landscape—at the borders between agricultural and other land. Wasp nests were said to be currently found almost exclusively in forests, a notable change within living memory. This was often attributed to increased use of agrochemicals, including pesticides, presumably because these harm the wasps and/or their insect prey. Some interlocutors explicitly discussed many insect species’ important ecological roles and their population levels as good indicators of an ecosystem’s health, and expressed concerns about the impacts of overharvesting, decreased diversity and changes in farming methods. Despite these observations and strong opinions, many of the same interlocutors used pesticides on their own crops.

Annual variations in weather may also have an impact on wasp populations. One carer in November 2014 described how the previous year he had kept ten nests, while that current year he had only four, which he attributed to the particularly wet spring which had ruined many nests in the ground.

Several interlocutors remarked on the areas within the forest in which wasp nests are commonly found. They are said to thrive in “healthy” areas of forest, with greater species diversity and undergrowth cover. Wasp searches tended to begin in such areas. New queens were also released into such areas, as these surroundings are considered to foster the best chances for the colony to be initiated and grow. Interlocutors working in the forestry industry, who also collected wasps for food, agreed and saw their work in forest management as supporting wasp conservation.

#### Cultural significance of wasp–human relations

People consider wasps to be a healthy food, rich in essential nutrients, and a good protein source. Several interlocutors also voiced beliefs that eating wasps can aid stamina and belong to a larger category of ‘virile’ foods. This is also reflected in the predominance of men in chasing, collecting, and caring for the wasps. Women may have been more involved in the hunt and collection in the past, at a time when mountain resources such as firewood, edible plants, and mushrooms were more integral to everyday life. We encountered only a single couple who still regularly chase wasps as a duo, but heard from the wives of collectors that sometimes they were called to help their husbands find nests. Reportedly, women were most often involved in the role of getting the wasps to take the bait, a division of labour still practised by the couple we observed. Processing and cooking them is most commonly done by women, as is the case for most food processing and preparation in the communities in which we worked.

A notable example of the identification of wasps with male virility was a dinner hosted by the exclusively male Vespula Society in Asuke, Aichi Prefecture, in November 2014. CP and JE were welcomed to the table, where wasp larvae, giant hornets (larvae, pupae, adults, and liquor infused with the adults), wild deer, matsutake mushrooms, and wild mountain yam were presented as a “feast” (ごちそう/*gochisō*) of “viagra” (ビアグラ/*biagura*). The classical/Renaissance Doctrine of Signatures, in which an organism’s medicinal function may be deduced by its resemblance to certain human body parts (eg. walnuts being good for the brain), may be at work here, evident in the penetrating ‘sting’ of the wasps and hornets, alongside the phallic matsutake mushrooms and mountain yams [56, 57]. The virility associated with eating the wasps is also closely related to the prestige of being skilled at finding and caring for them. In Kushihara, for example, a now-retired pre-eminent wasp carer is frequently referred to by his peers as the “God of wasps” (ヘボの神様/*hebo-no-kamisama*).

Wasp carers are explicit about their emotional and embodied attachment to the wasps they care for. One carer, when asked what role wasps play in his life, said they were his “girlfriend(s)” (彼女/*kanojō*), while closing his hand into a fist and keeping his little finger open—a lewd gesture in Japan used to denote sexual relations with women. Another had placed a chair in front of his hive boxes, and would sit and watch the wasps flying to and fro every morning. This may be a common practice, as carers will often try to predict nest size based on the degree of activity of wasps leaving and returning to the hive. The affection held by many wasp carers is further reflected in the names of village-level wasp societies, several of which are suffixed 愛好会 (*aikōkai*), which literally means ‘loving group’. It is clear that many carers have intimate knowledge of, and affection for, their hives.

The practice of overwintering new queens requires further physical care and attention, even to individual wasps. Some collectors reserve selected nests for this practice, and do not harvest the larvae. They place short, hollow shafts of bamboo along eaves, shelves and other surfaces near their selected hive boxes. Recently-hatched and newly-mated new queens choose these dark, narrow hollows as hibernation sites. During the hibernation period, the carer will transfer each new queen to a custom-made protective box, on a daily basis. Boxes each hold up to one hundred wasps, and are prepared with dry leaves, an air hole, and some source of food and water in case they awake early. Wasps are carefully transferred using handheld tweezers. The boxes are then placed under a covering on the land of the wasp collector, who assumes that this care will give the new queens greater protection from environmental hazards than a hibernation site in the forest.

A less time-intensive method also hoped to enhance new queen survival over winter is to leave one or two nests unharvested. One carer who does so explained that he has not overwintered new queens for over four years, because “it takes a lot of time, to keep and store them.” Instead, he stops giving food to the selected hive(s) after September, so the wasps return to eating a varied diet from the surrounding environment to “make them ready to produce good queens that will hibernate well over winter and build good colonies the next year.”

### What have been the key developments in wasp–human relations?

The timeline in Table 3 shows that key developments in wasp–human relations occurred in stages, driven by local innovations that became adopted in neighbouring communities. The first stage occurred when people began to keep wasps in hive boxes, the earliest record of which dates from 1916 [47]. A second stage was in 1990 when wasp care was first coordinated as a collaborative activity by members of one community, who established a purpose-built house for keeping multiple wasp hives. The same year saw the first wasp nest contest and media attention. A third stage occurred in 1994, when one individual first successfully kept new queens over winter, enabling people to extend their care of wasps to 11 months of the year [47].

**Table 3:** Timeline of developments in wasp-rearing practices from 1916-2013

Year	Development
1916	Keeping wasps in hives over the summer months: The first known mention of the use of hive boxes to keep social wasps appears in a magazine.
1990	Collaborative raising of multiple nests in a single purpose-built house: The first known ‘hebo house’ is built and used in Ishino, Aichi prefecture.
	Competitive showcasing of reared nests and information exchange: The first wasp nest contest is held in Shitara, Aichi prefecture. This was also broadcast on television and reported in national newspapers.
1994-1995	Keeping hibernating queens in protective boxes over the winter months: First known to be done successfully by Miyake Naome in Kushihara.
	Nationwide wasp contest begins: Held in Kushihara, attracting both local and non-local participants. 1994: Winning nest 2.9 kg, 52 participants 1995: Winning nest 3.6 kg
1997	Nationwide Japan Vespula Society founded: The first annual summit meeting held in Kushihara, with 12 groups from 5 prefectures. Participants shared information and experiences regarding wasp care.
2006 -2013	Results of Kushihara wasp nest contests suggest rearing practices have led to increases in the weight of the winning nests and in contest participants, compared to the early contests (there is not sufficient data from earlier years to test whether this difference is significant): 2006: Winning nest 7.7 kg. 2008: Winning nest 7.8 kg, average 1.9 kg, 140 participants 2009: Winning nest 6.0 kg, average 2.1 kg, 137 participants 2011: Winning nest 6.0 kg, average 2.2 kg, 140 participants 2012: Winning nest 6.5 kg, average 2.2 kg, 95 participants 2013: Winning nest 5.0 kg, average 1.8 kg, 130 participants

**Fig. 8:**
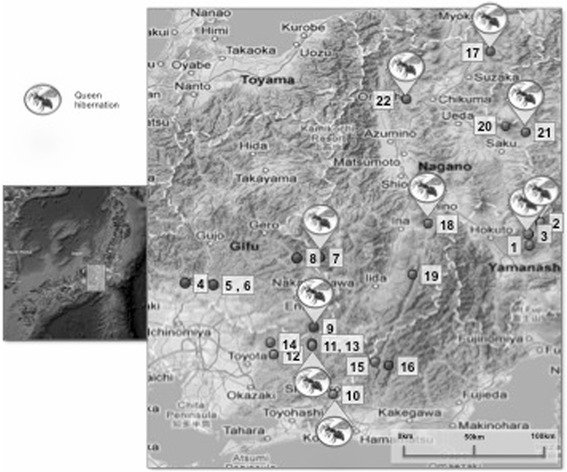
Map showing the location of known community groups and the prevalence of the queen hibernation in 2010 (the most recent meeting of the 全国地蜂サミット/*zenkoku-jibachi-samitto*). Numbers correspond to place names detailed in Appendix 1

These developments may have increased yields of wasp larvae, as suggested by the results of wasp nest contests listed in Table 3, which attest to increases in numbers of participants in contest, and in the size of the winning nest. Furthermore, a network of information exchange created by the establishment of the Japan Vespula Society (全国地蜂連合会/*zenkoku-jibachi-rengōkai*) in 1997 has also led to an increase in these practices throughout rural central Japan. In 2010, this network consisted of 22 community interest groups of which at least 10 practiced queen hibernation, as shown in the map in Fig. 8 (see Appendix 1 for place names).

### How much time do people in contemporary Japan devote to finding, collecting and keeping wasps, and how many wasp nests do they keep per person?

Questionnaire responses (*N* = 73) are shown in Table 4, and these suggest that the average wasp carer participates in 10.4 chasing trips in spring, and keeps seven nests in hives over the summer months. Each chasing trip is assumed to span approximately 8 h, involving baiting and following the wasps on foot through mountainous terrain. This was observed to be common practice during fieldwork in 2013–14 and was supported by follow-up interview data. Each nest is assumed to represent a once- to twice-daily investment in time and money, due to the observed common practice of feeding hives with raw meat and fish.

**Table 4:** Average reported time spent wasp-chasing, number of nests found and reared in hives, and what people chose to do with the wasp harvest, as answered by self-defined wasp chasers

					What do you do with the wasp larvae that you harvest in Autumn?			
	N	How many wasp hunting trips during spring 2013?	Average number of nests found	Average number of nests kept in hives	Share with family %(SD)	Give to friends in the neighbourhood %(SD)	Give to others %(SD)	Sell %(SD)
Kushihara	10	10.4	11.3	6.7	56 (±32.4)	37 (±33.5)	24 (±21.9)	4 (±10.1)
Ina	26	11.7	8.9	5.1	64 (±28.3)	37 (±15.5)	27 (±22.2)	15 (±21.2)
Higashi-shirakawa	37	9.5	12.8	8.4	39 (±29.3)	33 (±28.8)	18 (±23.8)	9 (±15.8)
Total	73	10.4	11.2	7	50 (±31.2)	34 (±26.8)	21 (±23.1)	8 (±14.9)

The activities of one wasp carer during a 10-year period are shown in Table 5. He spent an average of 31.2 days per year chasing wasps, in groups averaging three members, with an average harvest of just 0.4 nests per person per trip. He overwintered an average of 2543.3 new queens per year.

**Table 5:** The personal records of an individual wasp collector in Akechi, Ena city, Gifu prefecture

Year	No of trips	Average group size	Nests collected	Nests per trip	Nests per person	Nests per person per trip	Wasps hibernated
2002	37	4.28	69	1.9	16.1	0.4	2146
2003	39	3.9	79	2.0	20.3	0.5	3257
2004	42	3.63	96	2.3	26.4	0.6	1903
2005	41	2.46	53	1.3	21.5	0.5	1765
2006	29	2.66	28	1.0	10.5	0.4	5800
2007	22	2.95	19	0.9	6.4	0.3	1118
2008	29	2.72	23	0.8	8.5	0.3	2255
2009	25	2.64	20	0.8	7.6	0.3	2313
2010	26	2.69	12	0.5	4.5	0.2	2045
2011	24	2.59	13	0.5	5.0	0.2	3145
2012	29	2.52	10	0.3	4.0	0.1	2229
Average	31.2	3.0	38.4	1.1	11.9	0.4	2543.3

For each year: ‘Number of trips’ indicates the total wasp chasing trips that he was a part of during the spring; ‘Average group size’ indicates the average number of people with whom he went on each trip; ‘Nests collected’ indicates the total nests collected by groups that he was a part of; ‘Wasps hibernated’ indicates the total number of wasps that he collected and put into protective boxes in the late autumn, for hibernation over the winter months

### What reason do people give for starting to keep wasps, and what do they do with their harvest?

Of the questionnaire participants who responded to ‘Why did you start keeping wasps?’(Table 6) (*N* = 47), most referred either to enjoyment (*N* = 16) or to the influence of tradition/parents (*N* = 15). Such sentiments were also voiced in interviews. The head of the Kushihara Wasp Loving Society (串原ヘボ愛好会/*Kushihara -hebo -aikōkai*), for example, said he collects “as a hobby—as some people like to fish.” He also drew satisfaction from encouraging and supporting other people to care for wasps and trying to develop the wasp–human community: “I do it for everybody.”

**Table 6:** Summary of the responses to the open question asked to self-defined wasp-chasers (Questionnaire B): ‘Why did you start keeping wasps?’ Example responses from each category are given in the final two rows (in both Japanese and English)

		Why did you start keeping wasps?				
Wasp festival	N	Tradition/Influence of parents	For use as food	Enjoying nature	Enjoyment	Influence of non-relatives
Kushihara	10	2	1	2	4	0
Ina	26	9	1	1	4	1
Higashi-shirakawa	37	4	2	5	8	3
Totals	73	15	4	8	16	4
Example response (Japanese)		*子供の頃からやっていたから (kodomo no koro kara yatte ita kara)*	*食用の為 (shoko yō no tame)*	*山が好き (yama ga suki)*	*楽しそう (tanoshisō*)	*回りの友達 (mawari no tomodachi)*
Example response (English)		*Because I’ve done it since I was a child*	*For use as food*	*I love the mountains and forests*	*It looked like fun*	*Friends around me*

No interlocutors expressed a financial motivation for the practice. Survey respondents reportedly only sold an average of 8% (±14.9%) of their harvest for profit, shared 50% (±31.2%) with their family, and shared a further 34% (±26.8%) with people in the neighbourhood (Table 4).

## Discussion

### Care

The practices we observed in Japan do not fit neatly with concepts of ‘keeping’, ‘rearing’, and ‘farming’, all of which over-emphasize human agency and undervalue that of the wasps. Care Theory, developed by scholars across Science and Technology Studies, gives us a more accurate terminology [58, 59]. Care involves attentiveness and responsiveness to both humans and non-humans, all of whom are participants in public life [60, 61]. This is a crucial part of human-wasp relations, which are embedded within a larger web that encompasses human social relations as well as other organisms in agricultural and forest ecosystems.

While Care Theory emphasises the nature of humans as relational rather than autonomous, it also acknowledges this “interconnectedness” [62] is not always symmetrical: “Practices of care are always shot through with asymmetrical power relations: who has the power to care? Who has the power to define what counts as care and how it should be administered?” [58] In the case of human–wasp relations, human practices of care are shaped by our perceptions and assumptions of what is best for wasps, even if they are not necessarily so. Taking care “doesn’t [necessarily] mean being in charge” [59], or even being right.

Care Theory opens up a space to investigate how humans and wasps affect each other in unexpected ways. It allows us to describe the assemblage of practices, knowledges, and tools humans use to chase, collect, care for, harvest, share, prepare, and eat wasps. It also provides a practical vocabulary to think through what a human–wasp domesticatory relationship and its concrete mechanisms might look like.

### How can we conceptualise different categories of insect care?

Based on the case of wasps in rural Japan and supported with examples of other insect species, we propose a framework for kinds of insect care, outlined in Table 7. These relationships of mutualistic resource use differ by degree from opportunistic harvesting, are non-hierarchical and may co-occur. Each relationship can be observed in behaviour and potentially discerned in evolutionary history. The relationships are not necessarily intentional, and there is no implied progression between stages.

**Table 7:** Practices related to wasp care in Central Japan, with examples of other relevant human–insect relationships. An earlier version of this table and its accompanying text was published by Payne (2015) in Japanese in the journal 生物化学 (Journal of Biological Sciences) [64]. A discussion of the meanings of Japanese verbs used here can be found in Appendix 2

Shorthand	Operationalised as	Main impact on	Practices specific to wasp care	Further examples of insect care
‘Harvesting’ 採集する (*saishū-suru*)	Structured/strategic/systematic harvesting	Population demography	(1) Harvesting that is influenced by human land use patterns (e.g. preferential harvesting from locations near to roads, settlements); (2) Harvesting that is limited by concerns about population preservation - e.g. some collectors decide to leave nests/areas untouched for fear of over-harvesting.	*Anaphe panda,* Democratic Republic of Congo [98]; *Gonimbrasia belina*, Namibia [99]; *Encosternum delegorguei,* Zimbabwe [100].
‘Provisioning’ 餌をやる (*esa wo yaru)*	Encouragement/cultivation/relocation of preferred food source	Nutrition and reproductive success; Distribution	Leaving food for wasps in locations that are anticipated to be close to nests, mainly when searching for wasps to keep	*Cirina forda/butryospermi,* Nigeria [101]; *Oxya* spp., Asia [102].
‘Keeping’ 飼育する (*shiiku-suru*)	Relocation/altering location for one generation	Distribution; Adaptation to habitat	Placing wasp nests in wooden hive boxes*,* usually (but not exclusively) in areas where nests are not commonly found - e.g. in the village, next to houses.	*Vespula* spp., Japan [103]; *Anaphe panda,* Democratic Republic of Congo [98].
‘Herding’/ ‘Ranching’ 養殖する (*yōshoku-suru*)	Maintaining relocation/altering location across successive generations	Altered selective pressure	*n/a*	In parts of DRC, people move caterpillar colonies to feeding trees nearer to the household [104]. Whether this altered location is then maintained over successive generations is unknown.
’Cultivating’ 養殖する (*yōshoku-suru*)	Maintaining relocation/altering location AND encouragement/cultivation/relocation of preferred food source throughout the organism’s life cycle and across successive generations	Altered selective pressure	Overwintering of gynes, and re-release within an enclosed area near hive boxes with the hope that a new queen begins a colony directly in a hive box, is a conscious attempt to achieve this, but has not yet met with success.	*Tenebrio molitor, Gryllus* spp.*,* the Netherlands [105]; *Acheta domesticus,* Thailand [69]. *Gonimbrasia belina*, Zimbabwe [71].

The term ‘harvesting’ is used here to describe insects that are harvested by humans in a structured, strategic, or systematised way. That is, people limit their harvesting, every season, in a consistent way. These limitations can include the timing and area of the harvest, as well as the identity of the harvesters. These kinds of practices are likely to have an impact on insect population demographics that differs by degree from the effects of random, opportunistic harvesting.

We use the term ‘provisioning’ for situations in which humans organise and/or cultivate an insect’s food source. This includes cases in which the food species is planted near to human settlement, and in which the preferred species is itself cultivated. These practices have the impact of altering the distribution of the insect species, and in the latter case, may have a positive impact on their nutritional status and reproductive success. The ecology and behaviour of larger animals change in response to provisioning [63], and similar trends may be found in the insect world.

The term ‘keeping’ is defined here as the practice of relocating a species to a novel, human-made environment for part or all of its life cycle. This removes the insect from its natural habitat, altering its distribution and potentially resulting in developmental adaptations. When people keep insects, it becomes clear that the ecology of the insects changes as a result: *Vespula flaviceps/Vespula shidai* wasps build larger nests, for example, and the cocoons of *Anaphe panda* caterpillars change in shape [64].

‘Herding’ and’ranching’ involve maintaining and/or altering a species’ location across multiple generations. For larger animals, ranching usually involves keeping animals on privately owned land, while herding/pastoralism refers to keeping animals on public or communal land [65]. We amalgamate these here, but acknowledge that this difference can lead to quite different relations between humans, animals, and landscape.

Our final category, insect ‘cultivating’, is the practice of keeping insects through successive generations. This is perhaps closest to satisfying the criteria for stricter definitions of domestication. However, since edible insect breeding is, with the exception of honeybees and silkworms, a fairly novel practice, genetic changes may not yet be discernible at this stage. Cultivating with non-random harvesting will lead to an alteration in selective pressures and eventually genetic change. Insects have a far shorter generation time than large mammals, and are thus likely to show signs of directed evolution at a faster rate. This is seen to some extent in different races of *Apis mellifera* [66]. When it comes to specific species, the suffix ‘-culture’ could become useful here, as in ‘apiculture’, ‘sericulture’, and potentially, ‘vespiculture’.

These five patterns of edible insect resource use have different ecological impacts and therefore pose different questions and concerns. The transition from traditional practices of insect care to intensified industrial production may be part of a greater trend towards agricultural intensification, but it is occurring at a later date in human history than for other plant and animal species. Insect farmers currently developing novel methods can thus learn from historical domesticatory relationships. Table 8 outlines questions and priorities for future research into the ecologies and cultures of insect care.

**Table 8:** Questions and priorities for future research into the ecologies and cultures of insect care. An earlier version of this table and its accompanying text was published by Payne (2015) in Japanese in the journal 生物化学 (Journal of Biological Sciences) [64]

Category of edible insect resource use	Research questions	Research priorities
Harvesting	How have people traditionally placed limits on the harvest of edible insects? What is the impact of traditional harvesting practices on annual population numbers?	Ethnographic studies of insect harvesting practices. Comparative studies on the ecology of tended vs. non-tended insect populations.
Provisioning	Do provisioned insects have greater reproductive success than non-provisioned insects? Do provisioned insects differ in nutritional composition compared to non-provisioned insects?	Comparative studies of the ecology and genetics of provisioned vs. non-provisioned populations. Analysis of the nutrient composition of provisioned and non-provisioned insects.
Keeping	Do kept insects show behavioural patterns not found in wild populations? Are wild insect populations depleted in areas that practice insect keeping?	Behavioural and genetic studies of wild and kept insects. Population density surveys of insects in areas with a tradition of insect keeping.
Herding/Ranching	Are there human communities where these practices involve insects as food? If so, has it altered the species’ ecology and/or genetics, and/or human ranging practices?	Studies of edible insects that are relocated over multiple generations by human communities in order to be harvested as food. Studies of genetic diversity of insect species that have been relocated over multiple generations by human communities.
Cultivating	Are inbreeding effects detectable in commercially raised insects? How can farmers limit the threat of species-specific disease?	Studies of genetic diversity in commercially raised insects. Studies of disease immunity in insect species bred for food and feed.

### What are the potential biological impacts and modes of selection in current wasp–human relations?

The potential biological impacts of wasp–human relations are shaped firstly by the factors that lead to one nest being selected over another, and secondly by the impacts of care on the overall reproductive success of selected colonies.

Factors that lead to a nest being selected for care appear to be directly related to the ease with which humans can locate nests, since every nest found in the forest is collected. Thus, nests that are close to roads and forest paths have a higher chance of being selected. There is a second selection when nests are chosen for overwintering. Collectors who practiced overwintering did not have clear criteria for choosing nests and simply ensured that they were active nests. However, they did have clear criteria when selecting nests to be harvested for wasp contests: they chose the nest they considered likely to be largest. Therefore the nests chosen for overwintering may represent a sample that excludes the largest nests, i.e. those that have responded most dramatically to human care. In this case, strategies of care may in fact harm the reproductive success of the colonies most responsive to human care.

The immediate impact that current practices have on wasp ecology is the relocation of the nest into close proximity with human landscapes. Such landscapes include agricultural crops, a rich source of insect food for the wasps. All carers provision their wasps, which gives them daily, reliable access to a high-energy food source that they can eat without traveling far from the hive. Thus the wasps can propagate and sustain a far larger colony of workers, males and new queens.

Relocation to the hive box also gives them a form of protection unavailable in the *yama*. This is an effective barrier to the small land mammals that are their major predators. Although a minority of colonies do die after relocation, it is unclear whether or to what degree this is a result of relocation. Those who practice overwintering believe that wasps that hibernate in boxes are more likely to survive the season than those left in the forest. Further study is required to investigate this assumption.

Overall, the practice of caring may select *against* wasp colonies that are more likely to be harvested by humans in established practice, and many carers recognise this possibility. They note fluctuations in the abundance of nests each year, and are concerned about over-harvesting. Strategies of overwintering, and of leaving one or two nests unharvested, are intended to counter these concerns. However, only a tiny minority of wasp collectors—at present, fewer than one in each wasp-collecting community we spoke with—practice overwintering. Overwintering certainly has the potential to increase the reproductive success of colonies that are most likely to be collected by humans, and most likely to survive in the environment of the hive and overwintering boxes. Yet this also assumes that humans know best how to enhance the new queens’ well-being.

To further understand the potential impacts of wasp–human relations on wasp biology would require a quantitative comparison of colonies that are cared for with those that are not—an excellent direction for future research.

### Is wild harvesting a practice that encourages people to conserve biodiversity?

The wasps described here fit the FAO definition of a Non-Wood Forest Product (NWFP), which “*include[s] all goods of biological origin, as well as services, derived from forest or any land under similar use, and exclude wood in all its forms”* [67]. There is ongoing debate in conservation science about whether using NWFPs helps or hinders conservation objectives. For example, “Under what conditions is trade in captive or wild-harvested species beneficial for wild populations of the traded species?” is considered one of the top 100 questions of importance to the conservation of global biodiversity [68].

In the current case, it is certainly true that wasp carers were concerned about species preservation and the effects of their actions on long-term wasp survival. This is most clearly shown by the practice of overwintering. Yet as discussed above, this practice was not widespread.

Furthermore, interlocutors acknowledged that increased pesticide use may be to blame for more restricted distribution of wasps in living memory, yet also used pesticides in their own fields. This disjunction between concerns and actions may be explained by the fact that caring for wasps was not an integral part of interlocutors’ primary identity. The subsection of Japanese society to which they belong has other allegiances: they are members of rural farming communities with limited land, who struggle to compete with increasingly cheap imports. Agrochemicals increase yields and reduce labour, enabling them to provide for themselves and their children. In rural areas, organic goods do not sell for higher prices, and organic farmers are in the minority. When asked about pesticide use, many individuals invoked wartime memories of food scarcity. The widespread introduction of agrochemicals after WWII seems to have created a strong allegiance to chemically-assisted agriculture among this age group. The importance of wasps does not override the importance of consistent agricultural productivity.

### How do wasps, and wasp–human relations, inform human identity?

The ways in which humans understand and interact with wasps intersects with wider notions of identity in central rural Japan, particularly those of gender, community, and national identity.

In rural Japan, all activities, from labour to recreation, tend to be explicitly gendered, and wasp–human interactions are no exception. Collecting the nests involves entering the *yama* environment; this in itself is considered risky, which is the reason given for it being designated a male task. Wasps are one among many *yama* foods associated with virility. However, the everyday nature of wasp care as a practice does not carry connotations of risk. Instead, actions such as replenishing the wasps’ food, sitting and observing the hives, and placing individual wasps into boxes for overwintering are both safe and monotonous, yet are still primarily considered part of the male domain. Their success depends less on skill or daring, and more on a combination of luck and commitment. The same can be said of other popular hobbies among the same generation of Japanese men, such as fishing and pachinko.

Wasp-human relations also constitute, to varying degrees, a significant part of community identity in some areas. This is evidenced by wasp-loving societies, the majority of which are named after the main village or area they represent. It is also evident in regionally-specific names given to the wasps themselves (*hebo*, etc.): the relationship between the community and the wasps is linguistically unique, and opaque to outsiders, to whom it must be explained. This community-specific association with wasps is sometimes capitalised on by shopkeepers and restaurateurs, who offer speciality dishes that use the local name for the wasps, such as ヘボ飯 (*hebo-meshi*, ‘wasp-rice’). This marketing strategy is used widely throughout Japan, particularly in rural areas as part of 村起こし (*mura-okoshi*), efforts to promote the village by attracting domestic tourism and a subsequent source of income to areas that otherwise suffer from a shrinking, aging population and lack of jobs.

Finally, the wasps themselves are often described as 大人しい (*otonashii*, gentle, meek, tame), when compared to other stinging insects (such as other species of wasp). This is a word that is used to describe the character of the Japanese honeybee in comparison to the European honeybee, the latter of which is said to be far more 攻撃的 (*kōgeki-teki*, aggressive). Furthermore, the word *otonashii* is also often used generally, in contexts unrelated to wasps, to describe the Japanese character. Whether either character has been shaped to any extent by human–wasp relations is an intriguing possibility for future study.

### How do linguistic differences affect the conceptualisation of domestication?

Linguistic differences in terminology associated with domestication can have important implications for which practices and politics of care are conceptually possible. Of key importance here is the lack of a Japanese equivalent word for ‘domestication’. Several Japanese terms that describe different kinds of human practices of care towards other beings are described with examples in Table 7 and Appendix 2. Further studies could ascertain to what degree these linguistic differences manifest as different attitudes towards multispecies relations in Japanese culture.

Understanding how different languages survey this conceptual territory is crucial. Ignoring linguistic nuances can lead English-language discourse to assume that the material reality of multispecies relationships outside its own geolinguistic zone must be correspondingly impoverished and rudimentary. The devastating effects of this obliviousness has unfolded in many colonial projects, for example in Australia [95], where Aboriginals’ sophisticated modes of food procurement and production were utterly overlooked or ignored by colonists, and whose remaining traces are only now being investigated.

### How does this case engage with the larger debates in domestication studies?

This case complicates some of the debates identified in section 1.

#### Did domestication emerge because of surplus or scarcity?

Contemporary human-wasp relationships are, from a human perspective, first and foremost for social, leisure and gastronomic reasons. Wasp care involves great investments of time, money, and energy, and average returns per person are low. Additional benefits include social esteem from giving wasps as a gift, social bonding and prestige from skill in wasp collection and care, and the enjoyment of wasp care practices. The majority of wasp carers were male and elderly, which is a growing demographic in contemporary Japan. Per-capita income and consumption in elderly households exceeds those in younger households, which suggests that this group has the surplus necessary for undertaking wasp care [69]. In this case, therefore, ‘surplus’ seems to have been the greater driver.

Some practices of wasp care have developed by an individual innovation that has spread throughout the community, most recently with the practice of overwintering new queens. The spread of knowledge of these innovations was facilitated by mass media, and has inspired geographically separated communities to exchange information and techniques regarding wasp care. This cross-pollination of knowledge was likely assisted by the rapid industrialisation and political consolidation of Japanese rural communities during the latter half of the twentieth century, which has made information exchange increasingly possible between previously independent communities [70].

These developments have occurred in a cultural context that values wasps as a food item, and many interlocutors report having received wasp larvae as a gift. Gift-giving has particular significance in Japanese society [71], and the social benefits accrued by the giver may thus explain why the majority of harvested wasp larvae is shared rather than sold for profit.

#### Are agencies in domestication processes symmetrical or asymmetrical?

Both wasp carers and wasps themselves display agency. While humans are able to relocate the wasp nest, the colony ultimately decides whether to stay in its new location and surroundings. The wasp colonies elicit significant time, money, and energy from their human carers. They also influence human culture as icons and symbols that capture the human imagination and shape perceptions of gender, community, and national identities in human society.

#### Does domestication requires deliberation/intentionality?

An intention to domesticate does not seem to be part of these wasp–human relations. Overwintering of new queens illustrates the complexity of the deliberation/intentionality requirement. We observed clear deliberation among those human carers who overwinter new queens, yet none of the carers expressed that the goal of their deliberate action was hereditary reorganisation. Rather, inserting themselves between the stages of the wasps’ life cycle—their *interest* [72]—was, as they expressed it, to help ensure that there would be wasp nests to be found the following season, and the season after that. These practices of care may be and likely are having impacts on selection and adaptation within local wasp populations, but it is not carers’ expressed intention.

#### Does domestication entail a set of universal conditions that identifies all domesticates regardless of context?

Most literature on domestication focuses on a known set of vertebrate animals and plants. Yet some aspects of vertebrate domestication—hereditary reorganisation through herding and ranching, for example—may not apply to social insects. The operational generational unit for social wasps is the colony, which passes through an evolutionary bottleneck of the queen and the males with whom she has mated. This may result in evolutionary patterns that differ considerably from large vertebrate domesticates, for whom the generational unit is the individual organism.

#### Does domestication presume a converse state in which organisms are not already participating in mutual hereditary reorganisation?

Before humans first crossed from mainland Asia to the Japanese archipelago, *Homo sapiens* and *Vespula flaviceps/shidai* likely had no direct effect on one another. Today, even wasp colonies in remote parts of the Japanese mountains that are never harvested by humans will now be affected by anthropogenic climate change. Similarly, humans in wasp territories are affected by wasps in local ecologies and agricultures—their carnivorous predation of crop pests is but one example. This state of entanglement, now more than ever, is the backdrop against which all multispecies investigations take place. Wasps and humans in particular are already reorganising each other’s behaviours and have been doing so demonstrably for decades, if not centuries. What remains to be ascertained is the extent to which some of these reorganisations have become hereditary.

## Conclusions and recommendations for further research

In this paper, we have characterised the processes and practices of human–wasp relations in rural Japan, and we have investigated how this case informs debates in domestication studies.

Our main findings are:

- enjoyment and social meaning are key aspects of contemporary human–wasp relations in central rural Japan;
- the longer-term biological impacts of care are ambiguous, with potential for both adverse and beneficial impacts on wasp abundance and diversity;
- humans acknowledge their actions may both help and harm wasp survival, and contextual factors limit their commitment to wasp conservation;
- human perceptions of the wasps’ nature are in feedback with their self-perceptions;
- language plays a major role in shaping and perpetuating wasp–human relations; and
- this case contributes to several debates around domestication—in particular those of surplus versus scarcity as driving factors, active and passive roles, the importance and impact of intentionality, and the universality of domestication across species.

Overall, this case shows how a more nuanced conception of domestication as care can help us develop more convivial multispecies relationships, which are increasingly necessary in our changing environment. To further investigate the questions raised here, we propose studies that explore the interplay of affective, linguistic, economic, genetic and ecological factors that shape these relationships, within and beyond domestication studies.

We end our study with a proposal for reframing the domestication of social insects. Unlike the large mammals generally considered domesticates, social insects construct their own self-contained domus, without humans’ help; and while other animals do modify their human-provided habitats to some extent, the complexity and particularity of social insects’ home-building practices suggest a difference in kind. In this way the hive-box is a domus within a domus, a collaboration between humans and wasps to create a more ideal living arrangement for both. It embodies their multispecies entanglement, and may thus serve as a useful model for understanding relations of care between other species as well. A species may also leave the domus, and live again without us—they may become feral, like honeybees and many others—reminding us that ‘being domesticated’ is not a final status that is achieved, but a relation that is continuously made and remade and sometimes also made otherwise, in unpredictable, open-ended ways.

## Appendix 1

**Table 9:** Wasp-related associations in the Central Region of Japan

Prefecture	No. on Map	Organisation name (English)	Organisation name (日本語)	Year founded
Yamanashi	1	Nirasakimenokoshi Association	韮崎めのこし会	1995
	2	Sutama Hebo Research Group	須玉ヘボ研究会	2002
	3	Akeno Hebo Appreciation Society	明野ヘボ愛好会	1999
		Nagasaka Town Hebo Appreciation Society	長坂町ヘボ愛好会	1996
		Hachi Appreciation Society	蜂 愛好会	
		Nirasaki Hachi Appreciation Society	韮崎蜂愛好会	
Nagano	4	Samizu Jibachi Apiculture Research Group	三水地蜂養蜂研究会	1997
	5	Komoro Jibachi Appreciation Society	小諸地蜂愛好会	2003
	6	Omachi Jibachi Appreciation Society	大町路蜂愛好会	2002
	7	Ina City Jibachi Appreciation Society	伊那市地蜂愛好会	1997
	8	Heisei Group	平成会	1989
	9	Shinshu Hachi Appreciation Society	信州蜂愛好会	1994
		Ogawa Village Jibachi Appreciation Society	小川村飯綱地蜂愛好会	
		Kitaharaji Hachi Appreciation Society	北原路蜂愛好会	
		Matsumoto Sugare Group	松本スガレの会	
Gifu	10	Higashishirakawa Takabu Research Group	東白川タカブ研究会	1996
	11	Tsukechi Black Bee Club	付知ブラックビークラブ	2000
	12	Kushihara Hebo Appreciation Society	串原ヘボ愛好会	1993
	13	Seki City Hebo Appreciation Society	関市ヘボ愛好会	2000
	14	Nihon Heisei Mura Hebo Appreciation Society	日本平成村ヘボ愛好会	1997
	15	Yamagata City Hebo Club	山県市ヘボ同好クラブ	1996
		Yamadera Hebo Club	山寺ヘボクラブ	
		Kawabe Hebo Appreciation Society	川辺ヘボ愛好会	1996
		Ijira Hebo Club	伊自良ヘボ同好クラブ	1997
Shizuoka	16	Sakuma Hebo Appreciation Society	佐久間ヘボ愛好会	2000
		Ichima Haibachi Appreciation Society	笹間ハイバチ愛好会	1995
		Okabe Hebo Appreciation Society	岡部ヘボ愛好会	
Aichi	17	Ebi Hachi Appreciation Society	海老蜂愛好会	1999
	18	Tōei Hebo Group	東栄ヘボ会東栄ヘボ会	2004
	19	Kobakuro Research Group	こばくろ研究会	
	20	Asuke Jibachi Appreciation Society	足助地蜂愛好会	2001
	21	Ishino Community Hall Jibachi Group	石野交流館地蜂グループ	1990
	22	Fujioka Hebo Appreciation Society	藤岡ヘボ愛好会	2002
		Nagura Hebo Group	名倉ヘボグループ	
		Hōrai Town Hebo Appreciation Society	鳳来町ヘボ愛好会	1993
		Shitara Town Hebo Group	設楽町ヘボグループ	

## Appendix 2

### A discussion of the meanings of Japanese verbs used in Table 7

(Table 7 in our manuscript lists practices related to wasp care in Central Japan, with examples of other relevant human–insect relationships. An earlier version of the table and its accompanying text was published by Payne (2015) in Japanese in the journal 生物化学 (Journal of Biological Sciences) [57]. The following clarifications explain the meaning of the Japanese terms used in greater detail.)

採集する (*saishū-suru*) (‘harvesting’)

The first character of the Japanese verb 採集する (*saishū-suru*) does not have a meaning on its own, but the second character means ‘assemble’ or ‘gather’, and together they describe the action of collecting or gathering, e.g. shells, shellfish, insects.

餌をやる (*esa wo yaru*) (‘provisioning’)

The Japanese phrase 餌をやる (*esa wo yaru*) combines *esa*, animal feed, with *yaru*, the verb ‘to do’, and is used to refer to giving food to pets or livestock.

飼育する *(shiiku-suru*) (‘keeping’)

The Japanese verb 飼育する *(shiiku-suru*) combines the character for ‘keep’ (as in keeping animals) with the character for ‘growth’, and is used to describe the action of keeping, rearing, raising or breeding animals, in the context of both livestock and zookeeping.

養殖する (*yōshoku-suru*) (‘herding’ or ‘ranching’)

Similarly to *saishū-suru* above, the second character of the Japanese phrase 養殖する (*yōshoku-suru*) does not have a meaning on its own, though the first means ‘support’ or ‘maintain’, and together they describe cultivation or farming, usually of marine resources such as fish or oysters (other words are used for land agriculture and animal husbandry).

## References

[1] Bokonyi S, Clutton-Brock J (1989). Definitions of animal domestication. The Walking Larder: Patterns of Domestication, Pastoralism, and Predation.

[2] Ducos P, Clutton-Brock J (1989). Defining domestication: a clarification. The Walking Larder: Patterns of Domestication, Pastoralism and Predation.

[3] Smith BD (2001). Low-Level Food Production. J Archaeol Res.

[4] Cassidy R, Mullin M (2007). Where the Wild Things Are Now: Domestication Reconsidered.

[5] Russell N (2002). The wild side of animal domestication. Soc Anim.

[6] Anderson K (1997). A walk on the wild side: a critical geography of domestication. Prog Hum Geogr.

[7] Haraway DJ (1991). Simians, Cyborgs and Women: The Reinvention of Nature.

[8] Ingold T, Strathern M (1995). Building, dwelling, living: how animals and people make themselves at home in the world. Shifting Contexts: Transformations in Anthropological Knowledge.

[9] Latour B (1993). We Have Never Been Modern.

[10] Zeder MA (2009). The Neolithic macro-(r)evolution: macroevolutionary theory and the study of culture change. J Archaeol Res.

[11] Davis SJM (2005). Why domesticate food animals? Some zoo-archaeological evidence from the Levant. J Archaeol Sci.

[12] Isaac E (1970). Geography of Domestication.

[13] Hayden B (2003). Were luxury foods the first domesticates? Ethnoarchaeological perspectives from Southeast Asia. World Archaeol.

[14] O’Connor T (1997). Working at relationships: another look at animal domestication. Antiquity.

[15] Schleidt WM, Shalter MD (2003). Co-evolution of humans and canids. Evol Cogn.

[16] Gon SM, Price EO (1984). Invertebrate Domestication: Behavioural Considerations. Bioscience.

[17] Morey DF (1994). The early evolution of the domestic dog. Am Sci.

[18] Wheeler WM (1910). Ants; their structure, development and behavior.

[19] Mueller UG, Gerardo NM, Aanen DK, Six DL, Schultz TR (2005). The evolution of agriculture in insects. Annu Rev Ecol Evol Syst.

[20] Leach H (2003). Human Domestication Reconsidered. Curr Anthropol.

[21] Hare B, Wobber V, Wrangham R (2012). The self-domestication hypothesis: evolution of bonobo psychology is due to selection against aggression. Anim Behav.

[22] Bettinger R, Barton L, Morgan C (2010). The origins of food production in north China: A different kind of agricultural revolution. Evol Anthropol.

[23] Bharucha Z, Pretty J (2010). The roles and values of wild foods in agricultural systems. Philos Trans R Soc Biol.

[24] Scott J (2011). Four Domestications: Fire, Plants, Animals, and… Us. Tanner Lectures on Human Values.

[25] Smith BD (2007). Niche construction and the behavioural context of plant and animal domestication. Evol Anthropol.

[26] Odling-Smee FJ, Laland KN, Feldman W (2013). Niche construction. Monographs in Population Biology. Vol. 37.

[27] DeFoliart GR (1995). Edible insects as minilivestock. Biodivers Conserv.

[28] Johnson DV, Durst PB, Johnson DV, Leslie RN, Shono K (2010). The contribution of edible forest insects to human nutrition and to forest management. Forest insects as food: humans bite back. Proceedings of a workshop on Asia-Pacific resources and their potential for development.

[29] Chávez-Moreno CK, Tecante A, Casas A (2009). The Opuntia (Cactaceae) and Dactylopius (Hemiptera: Dactylopiidae) in Mexico: a historical perspective of use, interaction and distribution. Biodivers Conserv.

[30] Gordon BL (1957). A domesticated, wax‐producing, scale insect kept by the Guaymí Indians of Panamá. Ethnos.

[31] Zhang C, Tang X, Cheng J (2008). The utilization and industrialization of insect resources in China. Entomol Res.

[32] Kohler R (1994). Lords of the Fly: Drosophila Genetics and the Experimental Life.

[33] Hanboonsong Y, Jamjanya T, Durst PB (2013). Six-legged livestock: Edible insect farming, collection and marketing in Thailand.

[34] Halloran A, Roos N, Flore R, Hanboonsong Y (2016). The development of the edible cricket industry in Thailand. J Insects Food Feed..

[35] Van Itterbeeck J, Van Huis A (2012). Environmental Manipulation for Edible Insect Procurement. J Ethnobiol Ethnomed.

[36] Meyer‐Rochow VB, Changkija S (1997). Uses of insects as human food in Papua New Guinea, Australia, and North‐East India: Cross‐cultural considerations and cautious conclusions. Ecol Food Nutr.

[37] Hope RS, Frost PGH, Gardiner A, Ghazoul J (2009). Experimental analysis of adoption of domestic mopane farming technology in Zimbabwe. Dev South Afr.

[38] Ghazoul J (2006). Mopane Woodlands and the Mopane Worm: Enhancing rural livelihoods and resource sustainability Final Technical Report.

[39] Fukuda T, Hi Y, Motoichi M (1961). Artificial food for Eri-silkworm raising. Agric Biol Chem.

[40] Chakravorty J, Gogoi M, Benno Meyer-Rochow V (2015). Cultural Attributes and Traditional Knowledge in Connection with the Rearing of Muga (Antheraea assama = assamensis) in the Dhemaji District of Assam, North-East India. J Insect Biotechnol Ser.

[41] Mbahin N, Raina SK, Kioko EN, Mueke JM (2010). Use of sleeve nets to improve survival of the Boisduval silkworm, Anaphe panda, in the Kakamega Forest of western Kenya. J Insect Sci.

[42] Ngoka BM, Kioko EN, Raina SK, Mueke JM, Kimbu DM (2007). (2007). Semi-captive rearing of the African wild silkmoth Gonometa postica (Lepidoptera: Lasiocampidae) on an indigenous and a non-indigenous host plant in Kenya. Int J Trop Insect Sci.

[43] Nonaka K (2009). Feasting on insects. Entomol Res.

[44] Chakravorty J, Ghosh S, Benno Meyer-Rochow V (2013). Comparative survey of entomophagy and entomotherapeutic practices in six tribes of Eastern Arunachal Pradesh (India). J Ethnobiol Ethnomed.

[45] Ying F, Xiaoming C, Long S, Zhiyong C, Durst PB, Johnson DV, Leslie RN, Shono K (2010). Common edible wasps in Yunnan Province, China and their nutritional value. Forest insects as food: humans bite back. Proceedings of a workshop on Asia-Pacific resources and their potential for development.

[46] 松浦 誠. スズメバチを食べる*:* 昆虫食文化を訪ねて. 北海道大学図書刊行会. 2002. (*Matsuura M. Suzumebachi wo taberu: Konchū shoku bunka wo tazunete. Hokkaido daigaku toshokankōkai.*)

[47] Makino S, Katsuhiko S (2005). Species compositions of vespine wasps collected with bait traps in recreation forests in northern and central Japan (Insecta, Hymenoptera, Vespidae). Bull For For Prod Res Institute Ibaraki.

[48] 野中健一 「昆虫食先進国ニッポン」 亜紀書房. 2008. *(Nonaka Kenichi. [Konchū shoku seishin kyoku nippon] Aki shoubou.)*

[49] Payne CLR (2015). Perception and Practice of Entomophagy in Central Rural Japan. Trans Asiat Soc Jpn.

[50] Goodisman MAD, Kovacs JL, Hoffman EA (2007). Lack of conflict during queen production in the social wasp *Vespula maculifrons*. Mol Ecol.

[51] Markowitz F (2006). Blood, soul, race and suffering: Full-bodied ethnography and expressions of Jewish belonging. Anthropol Humanism.

[52] Cronon W (1996). The Trouble with Wilderness: Or, Getting Back to the Wrong Nature. Environ Hist.

[53] Lorimer J (2015). Wildlife in the Anthropocene: Conservation after Nature.

[54] Kuroda Y (2016). The Impacts of Factor Inputs-Subsidies on the Agricultural Structural Transformation of the Rice Sector. Rice Production Structure and Policy Effects in Japan.

[55] 西尾亮平 「ヘボ(地蜂)騒動記 その生態と魅せられた人々」自刊. 1999. *(Nishio Ryōhei. [Hebo (jibachi) sōdōki sono seitai to miserareta hito bito] Ji kan.)*

[56] Bennett B (2007). Doctrine of Signatures: An Explanation of Medicinal Plant Discovery or Dissemination of Knowledge?’. Econ Hist.

[57] Foucault M (1970). The Order of Things.

[58] Martin A, Myers N, Viseu A (2015). The Politics of Care in Technoscience. Soc Stud Sci.

[59] Puig de la Bellacasa M (2011). Matters of Care in Technoscience: Assembling Neglected Things. Soc Stud Sci.

[60] Curry JM (2002). Care Theory and “caring” systems of agriculture. Agric Hum Values.

[61] Latour B (2004). Politics of Nature: How to Bring the Sciences into Democracy.

[62] Sevenhuijsen S. Interview with Selma Sevenhuijsen. Ethics Care. 2013. http://ethicsofcare.org/selma-sevenhuijsen/. Accessed 12 May 2016.

[63] Mysterud A (2010). Still walking on the wild side? Management actions as steps towards ‘semi-domestication’ of hunted ungulates. J Appl Ecol.

[64] Payne C (2015). The ‘domestication’ of edible insects. Seibutsukagaku (J Biol Sci).

[65] LaRoque O (2014). Revisiting distinctions between ranching and pastoralism: A matter of interspecies relations between livestock, people, and predators. Crit Anthropol.

[66] Ruttner F (2013). Biogeography and taxonomy of honeybees.

[67] Chandrasekharan C (1995). Terminology, definition and classification of forest products other than wood. Report of the International Expert Consultation on Non-Wood Forest Products.

[68] Sutherland WJ, Adams WM, Aronson RB, Aveling R, Blackburn TM, Broad S, … Dinerstein E. One hundred questions of importance to the conservation of global biological diversity. Conserv Biol. 2009;23:557–67. 10.1111/j.1523-1739.2009.01212.x 19438873

[69] Yashiro N, Hurd MD, Yashiro N (1996). The economic position of the elderly in Japan. The Economic Effects of Aging in the United States and Japan.

[70] Nakano T, Brown K (1970). Changing rural Japan. In: Norbeck F, Parman S, editors. The Study of Japan in Behavioural Sciences. Rice Univ Stud.

[71] Befu H (1986). Gift giving in a modernising Japan. Monum Nippon.

[72] Stengers I (2000). The Invention of Modern Science. Smith DW, translator.

